# Assessing genetic diversity and performing genome‐wide association studies (GWAS) using multiple marker types across desi, kabuli, and wild accessions in chickpea (*Cicer arietinum* L.)

**DOI:** 10.1002/tpg2.70149

**Published:** 2025-11-23

**Authors:** Pradeep Ruperao, Anil Kumar Vemula, Vinod Kumar Valluri, Sriswathi Manda, Rakesh K. Srivastava, Sean Mayes, Rajeev K. Varshney, Himabindu Kudapa

**Affiliations:** ^1^ Global Research Program‐Accelerated Crop Improvement International Crops Research Institute for the Semi‐Arid Tropics (ICRISAT) Hyderabad India; ^2^ Data Management and Analytical Support International Crops Research Institute for the Semi‐Arid Tropics (ICRISAT) Hyderabad India; ^3^ Centre for Crop and Food Innovation WA State Agricultural Biotechnology Centre Food Futures Institute Murdoch University Murdoch Western Australia Australia

## Abstract

Genetic diversity is a key aspect of the selection of superior genotypes in crop varietal improvement. Breeding activities in chickpea (*Cicer arietinum* L.) have successfully enhanced the genetic diversity by introducing variations from wild relatives and landraces. Such diversity was characterized by employing various molecular markers, including single nucleotide polymorphisms (SNPs), insertions and deletions (indels), presence/absence variations (PAVs), and so forth. These marker types through different studies provided different levels of genetic variations from single base to gene structure level. The use of multi‐marker types for diversity analysis and genome‐wide association studies (GWAS) represents a powerful strategy. In the current study, whole genome re‐sequencing data from 593 select chickpea genotypes representing desi, kabuli, and wild types, with over 21 million SNPs, 10 million indels, and 16,117 PAVs, were analyzed. This study demonstrated enhanced diversity in both desi and kabuli types, with wild accessions showing higher diversity compared to landraces. A more comprehensive understanding and broader range of genetic diversity within and between desi, kabuli, and wild accessions, as well as landraces, cultivars, and breeding lines, were captured. The identified novel alleles and gene variations through this analysis offer effective breeding strategies for key traits such as yield, and biotic and abiotic stress tolerance that can significantly contribute to chickpea improvement. Overall, this study highlights the importance of balanced population design, use of multiple marker types in identifying diverse gene pool, and novel marker‐trait associations for key/optimal traits paving the way for the development of more resilient chickpea varieties.

## Core Ideas

- Use of multiple marker (single nucleotide polymorphisms [SNPs], insertions and deletions [indels], and presence/absence variations (PAVs]) types offers an effective strategy for breeding programs to improve yields by providing valuable insights into genetic variation, especially in wild accessions, and understanding complex traits such as drought and disease resistance.
- The study highlights importance of using multiple marker types in genome‐wide association studies (GWAS) analysis for identifying potential marker‐trait associations related to key traits in chickpea.
- The current study contributes to the understanding of evolutionary dynamics and selective pressures acting on protein‐coding genes in distinct chickpea populations.
- The diverse gene pool and marker‐trait associations underlying important agronomical traits identified serve as valuable resources for enhancing chickpea productivity and resilience in breeding programs.

## Plain Language Summary

Genetic diversity plays a vital role in chickpea breeding programs, serving as the foundation for developing improved varieties. Recent genomics research efforts in chickpea have successfully enhanced genetic diversity in both desi and kabuli types. Wild chickpea species are particularly valuable as they contain greater genetic diversity and variations that can be utilized for crop improvement. Using multiple marker types (single nucleotide polymorphism, insertions and deletions, and presence/absence variations), this study reports diverse gene pool and significant marker‐trait associations underlying key agronomical traits. These molecular tools, along with other markers, help identify novel alleles from wild species. This comprehensive approach to exploiting genetic diversity and marker‐trait associations is essential for developing more resilient and productive chickpea varieties.

**Abbreviations**
ATP: adenosine triphosphate
DTF: days to flowering
DTM: days to maturity
FAD: flavin adenine dinucleotide
FarmCPU: fixed and random model circulating probability unification
F_IS_
: inbreed coefficient
F_ST_
: fixation index
GATK: genome analysis tool kit
GO: gene ontology
gPAV: gene presence and absence variation
GWAS: genome‐wide association studies
He: expected heterozygosity
Ho: observed heterozygosity
HSW: 100‐seed weight
Indel: insertions and deletions
MAS: marker‐assisted selection
MLM: mixed linear model
MTA: marker trait association
PA: private alleles
PCA: principal component analysis
PH: plant height
pi: nucleotide diversity
PVE: phenotypic variance explained
SNP: single nucleotide polymorphism
Ts: transition mutation
Tv: transversion mutation
WG1: wild group 1
WG2: wild group 2
YPP: yield per plant
λGC: genomic inflation factor

## 1 INTRODUCTION

Chickpea (*Cicer arietinum* L.) is a globally grown legume crop that holds great nutritional and economic significance. Developments in genomics, including the availability of high‐quality genome assemblies (Garg et al., 2022; Jain et al., 2013; Parween et al., 2015; Varshney et al., 2013), pangenomes, and whole genome re‐sequencing (WGRS) data (Khan et al., 2024; Varshney, 2016; Varshney et al., 2019; 2021), have revolutionized chickpea breeding programs (Bohra et al., 2022). There are two types of chickpeas, desi and kabuli, contributing around 80% and 20% of total production, respectively. These possess different phenotypic characteristics such as seed attributes, disease resistance, and adaptability (Roorkiwal et al., 2020; Thudi et al., 2016). Chickpea's wild counterparts also contribute to genetic diversity in breeding programs (Varshney et al., 2021). Assessing the genetic diversity between desi, kabuli, and wild accessions is important for improving chickpea yields under changing climate scenario.

Next generation sequencing has revolutionized genomics advancements in crops facilitating the use of several molecular markers such as single nucleotide polymorphisms (SNPs), insertions and deletions (indels), presence/absence variations (PAVs), and so forth, in assisting the selection of plants with desired traits to improve the efficiency and precision of crop breeding (Basu et al., 2018; Ruperao, Bajaj, et al., 2023, 2024). In chickpea, it is evident from earlier studies that molecular markers played a key role in describing genetic variations in intra‐ and inter‐populations (Roorkiwal et al., 2020). Moreover, through WGRS one can determine the variations in the genome of the parental lines of mapping populations for understanding the genetic architecture of different traits (Thudi et al., 2016). Studies have also revealed the use of wild relatives as valuable sources of new allelic forms for breeding future crops (Singh et al., 2022).

However, utilizing one marker type may limit information on complex interactions that involve multiple genetic loci leading to inaccurate conclusions (Korte & Farlow, 2013; Uffelmann et al., 2021). To address this limitation, recent studies have used multi‐marker types, such as SNPs and PAVs, in genome‐wide association studies (GWAS) (Zhao et al., 2020). This approach improves understanding of population features and diversity, strengthening GWAS for the identification of trait‐associated markers and new alleles (Istanbuli et al., 2024; Salgotra & Stewart, 2022). The present study was conducted to demonstrate the application of multiple marker types in GWAS analysis of chickpea. Multiple marker types derived from WGRS data of 593 selected chickpea genotypes representing desi, kabuli, and wild accessions were analyzed. Our analysis established enhanced diversity in both desi and kabuli types, with a higher diversity in wild accessions revealing the potential use of multiple marker types in GWAS analysis. Additionally, novel alleles and gene variations were identified for use in chickpea improvement. In summary, we provide a better understanding and knowledge on chickpea diversity through the use of multiple marker types in diversity analysis and GWAS.

## 2 MATERIALS AND METHODS

### 2.1 Accession selection—Optimal sampling size

Chickpea accessions with more than 10x coverage WGRS data were selected for the study, along with variant data from an earlier study (Varshney et al., 2021). The genetic distance was calculated using R “labdsv” package with the Euclidean and Manhattan methods. The common list of 480 (262 desi and 218 kabuli) accessions was further assessed with heterozygosity to select 200 most diverse accessions in each desi and kabuli set. Further, all 193 wild accessions with WGRS data were used for the analysis. A total of 593 accessions were used for the study (Table S1).

### 2.2 Sequence alignment and variant calling

Aligning the sequence data to the chickpea pangenome (https://cegresources.icrisat.org/cicerseq) with Burrows–Wheeler Aligner‐MEM v.0.7.17 (Li & Durbin, 2010), the variants called from the standard genome analysis tool kit (GATK) v4.2.0.0 pipeline (McKenna et al., 2010) were further filtered with GATK parameters (‐Window 4, ‐filter “QD < 2.0, FS > 60.0, MQ < 40.0,” ‐G_filter “GQ < 20”) similar to the previous study (Varshney et al., 2021). The SNPs and indels were filtered and analyzed separately with the SelectVariants program of GATK pipeline and furthered with “m2 M2, F_MISSING < 0.2, MAF > 0.05” with bcftools v 1.9. Following SNP calling, snpEFF (Cingolani et al., 2012) was used for SNP annotations. Positive selection (or diversifying selection) refers to genes where the rate of non‐synonymous substitutions (dN) is greater than synonymous substitutions (dS) (i.e., *ω* > 1), suggesting that changes in genome sequence are favored and may confer adaptive advantages.

Negative (purifying) selection occurs when dN < dS (i.e., ω < 1), indicating that non‐synonymous changes are largely deleterious and thus eliminated by natural selection to preserve gene function, whereas the neutral selection is when dN ≈ dS (i.e., *ω* ≈ 1), suggesting the sequence changes are neutral and accumulate through genetic drift.

**Core Ideas**

- Use of multiple marker (single nucleotide polymorphisms [SNPs], insertions and deletions [indels], and presence/absence variations (PAVs]) types offers an effective strategy for breeding programs to improve yields by providing valuable insights into genetic variation, especially in wild accessions, and understanding complex traits such as drought and disease resistance.
- The study highlights importance of using multiple marker types in genome‐wide association studies (GWAS) analysis for identifying potential marker‐trait associations related to key traits in chickpea.
- The current study contributes to the understanding of evolutionary dynamics and selective pressures acting on protein‐coding genes in distinct chickpea populations.
- The diverse gene pool and marker‐trait associations underlying important agronomical traits identified serve as valuable resources for enhancing chickpea productivity and resilience in breeding programs.

Principal component analysis (PCA) was performed using PLINK 1.9 (Zheng et al., 2012). The first three dimensions were used to distinguish population structure, with each marker type analyzed independently. Using SNP markers, with Ape package (Paradis et al., 2004), the phylogenetic tree was constructed according to the neighbor‐joining method with iTOL (Letunic & Bork, 2019).

### 2.3 Functional enrichment analysis of variable genes

The variable genes with the associated gene ontology (GO) were analyzed to understand the approximate functions of these genes. All candidate genes were mapped to terms in the GO, and the number of significant genes for each term was determined with a *p* value ≤ 0.05 as a threshold. Terms satisfying this criterion were defined as GO terms that were significantly enriched for candidate genes.

### 2.4 Gene PAV detection

In order to further understand the genetic relationship between the accessions, a neighbor‐joining algorithm was used to construct a phylogenetic tree using the filtered SNP set. The gene PAVs were defined based on a minimum coverage of at least two sequence reads mapped to the respective chickpea pangenome genes with a frequency of 0.70, similar to the PAV pipeline used in sorghum pangenome (Ruperao et al., 2021; Ruperao, Gandham, et al., 2023). Based on the sequence reads mapping, a gene PAV matrix was generated showing the presence or absence of each gene in the respective accession. The genes present in all accessions were considered as conserved/core genes, the genes present in few accessions are variable/dispensable genes, and the genes present/absent in only specific accessions are the private (unique) genes. Using the Fisher's exact test, the statistical significance of gene frequency was determined. The *p*‐value < 0.001 and a minimum of 10% of the desi and kabuli type differences were considered significant.

To investigate genetic differentiation between desi and kabuli chickpea sets, vcftools software was employed to calculate the fixation index (F_ST_) for each SNP that remained after filtering. The genome‐wide distribution of F_ST_ values was then characterized, and the standard deviation was utilized to establish upper and lower threshold boundaries. SNPs exhibiting F_ST_ estimates beyond these thresholds were considered highly diverged between the two populations. By cross‐referencing these outlier SNPs against an annotated gene list, genomic regions and candidate genes putatively undergoing diversifying selection were identified.

### 2.5 Diversity analysis

Pairwise linkage disequilibrium (LD) was estimated between markers on the same chromosome using the *r* ^2^ statistic (Tang et al., 2023). The *r* ^2^ values were plotted against the physical distance of trait‐associated markers to visualize LD decay. The nucleotide diversity (*π*) measure was calculated to assess genetic variation within the population. F_ST_ was computed to quantify population differentiation due to genetic structure. Both observed and expected heterozygosity were calculated to evaluate genetic diversity, which indicates the proportion of individuals possessing two different alleles at a given locus. The inbreeding coefficient (F_IS_) metric was determined to assess the level of inbreeding within the population. All calculations were performed using vcftools (Danecek et al., 2011), a widely used software for analyzing genetic variation data.

### 2.6 Genome‐wide association studies

The GWAS analysis was conducted utilizing filtered SNPs, indels, and gene presence and absence variations (gPAVs) from the current study and mean phenotypic data of two field trials conducted at the International Crops Research Institute for the Semi‐Arid Tropics (ICRISAT) from Varshney et al. (2021). Marker‐trait association (MTA) analysis was subsequently performed using the fixed and random model circulating probability unification (FarmCPU) and mixed linear model (MLM) methods within the rMVP package (Yin et al., 2021) separately on each marker type. The default Bonferroni correction threshold was applied to control for multiple testing across the genome. This approach ensures that the reported associations are statistically robust and are not due to random chance. Manhattan plots, quantile–quantile plots, and *p*‐values obtained from the GWAS (both models) were evaluated using the threshold 0.05 to identify significant MTAs. Genomic inflation factor (λGC) is calculated with an in‐house developed script, and the lower λGC indicates a stable and possibly biologically meaningful signal, while higher λGC reflects noise or population structure effects.

## 3 RESULTS

### 3.1 Advances beyond previous studies

The current study represents a significant advancement over earlier findings as briefly presented below:

#### 3.1.1 Balanced population design

Utilization of an evenly distributed set of 593 chickpea accessions, addressing the limitations posed by uneven sampling in prior studies.

#### 3.1.2 Multiple variant type analysis

A comprehensive genetic diversity and GWAS analysis incorporating SNPs, indels, and gPAVs, providing a broad and multi‐faceted understanding of diverse gene pools and their genetic associations with agronomic traits.

#### 3.1.3 Novel MTAs

Identification of 39 previously unreported MTAs, underscoring the enhanced discovery achieved through use of multiple marker types for GWAS analysis.

#### 3.1.4 Comparative genomic insights

Detailed analyses of pan‐genome composition and diversity metrics, facilitated by the balanced sampling strategy.

### 3.2 Optimal sampling strategy and genetic diversity analysis

The population size plays a crucial role when comparing genetic diversity between different sets of samples (Ruperao, Bajaj, et al., 2023; Ruperao, Rangan, et al., 2023). To ensure an adequate sample size with higher diversity, approximately 200 accessions representing each of desi, kabuli, and wild types were selected from the 3366 chickpea accessions (Varshney et al., 2021). Hereafter, these different accession sets are referred to as populations. The accessions with more than 10x coverage, representing most diverse accessions based on the Euclidean and Manhattan genetic distance, and low observed heterozygosity (Ho) than the expected heterozygosity (He) were selected (Table S1).

**FIGURE 1:**
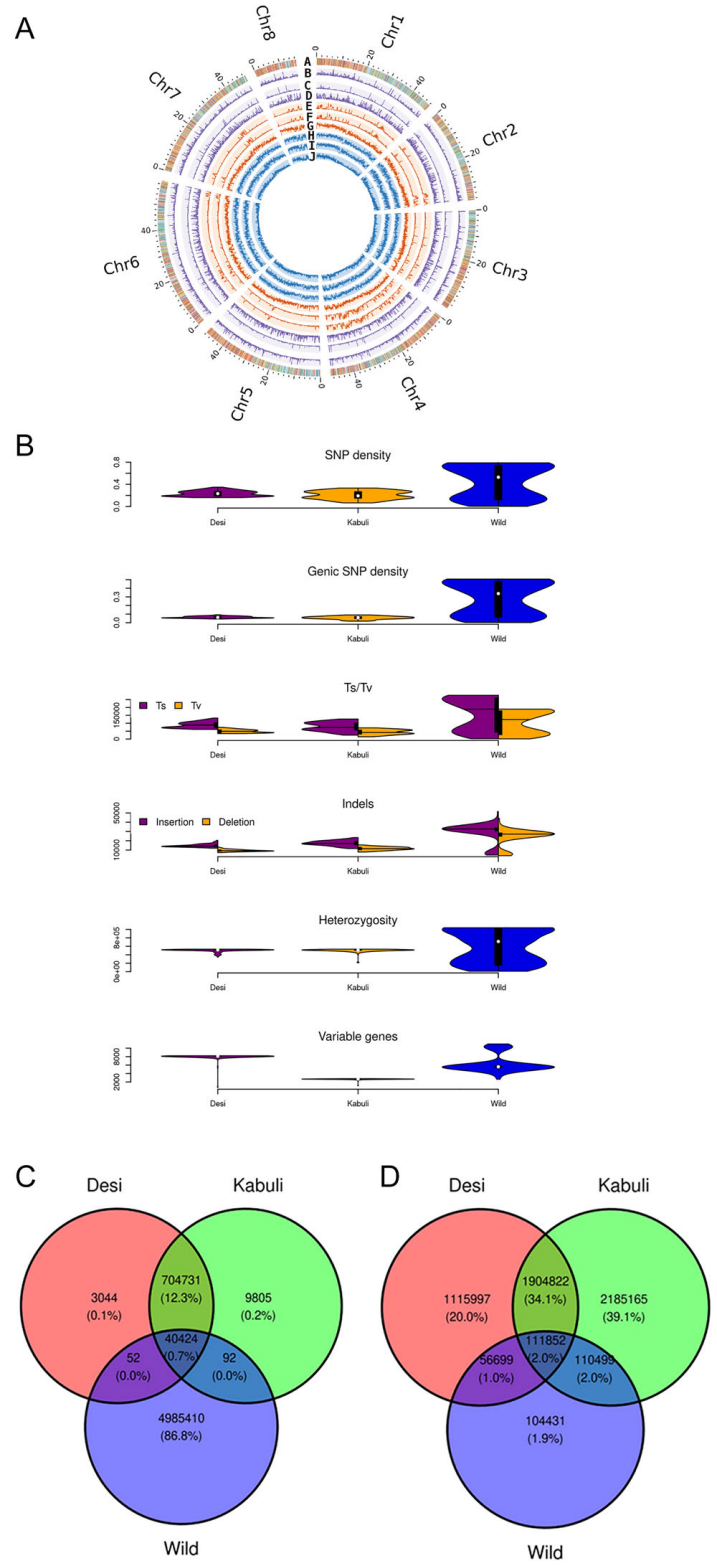
(A) The circos pangenome with the gene density on the outmost heatmap track (A), followed by the variable gene density (red background) in desi (B), kabuli (C), and wild populations (D); single nucleotide polymorphism (SNP) density (green background) in desi (E), kabuli (F), and wild (G); and insertions and deletions (indels) (blue background) in desi (H), kabuli (I), and wild (J) populations. (B) Plot shows the comparative SNP density, genic SNP, transition mutation/transversion mutation (Ts/Tv), indels, Hets, and variable genes between the chickpea populations studied. *Y*‐axis denotes “variants count” in the plot. The proportion of (C), SNP, and (D) indel count between chickpea desi, kabuli, and wild populations.

The aligned WGRS data to the reference genome identified 21,280,556 SNPs and 10,221,717 indels. To improve the accuracy of the analysis, we filtered these SNPs and indels by deletion of the loci not satisfying the filter parameters (discussed in Section 2) (Table 1). The number of filtered SNPs for desi, kabuli, and wild populations were 748,251, 755,052, and 5,025,978, respectively (Table 2). Molecular markers with an overall pi value 0.000645363, 0.000649127, and 0.00409278 were used for subsequent analysis (Figure 1A,B). High‐quality SNPs were obtained from three different groups, most of which were located in the intergenic regions for desi (729,268; 63.49%), kabuli (735,864; 63.47%), and wild (4,747,300; 42.51%) populations (Figure 1B). The exon (desi 2.83%, kabuli 2.84%, and wild 5.15%) and intron (desi 4.54%, kabuli 4.55%, and wild 8.35%) SNP counts differ comparatively in these populations. A comparative analysis of heterozygosity levels revealed that wild chickpea exhibited a considerably higher average (898,652) compared to both desi (504,003) and kabuli (503,842) accessions. The transition to transversion ratio in the desi (1.66) and kabuli (1.64) populations is nearly same, while the wild population has 1.39. Among different marker types, SNPs being more abundant, the private SNPs recorded in desi (0.1%) and kabuli (0.2%) populations are much less compared to the private indels reported in the chickpea populations studied (desi 20%, kabuli 39%, and wild 1.9%) (Figure 1C,D).

**TABLE 1:** Genome‐wide genes, filtered single nucleotide polymorphisms (SNPs), insertions, deletions, observed heterozygosity (Ho), and expected heterozygosity (He) count presented chromosome‐wise.

	Genes	SNPs	SNP density (kb)	Insertion	Deletion	Ho	He
Chr1	3060	646,328	13.36	302,767	301,436	214,518	155,255
Chr2	2231	465,607	12.71	237,296	238,121	150,405	108,711
Chr3	2759	551,244	13.78	257,432	259,628	172,834	126,786
Chr4	3469	834,937	16.97	334,002	331,223	303,079	210,671
Chr5	3165	612,876	12.72	287,362	289,790	189,694	139,715
Chr6	3764	757,477	12.74	372,014	373,236	241,997	177,346
Chr7	3096	653,966	13.36	313,320	314,097	209,093	150,909
Chr8	1439	294,733	17.89	125,457	123,404	93,307	67,053

**TABLE 2:** The total filtered single nucleotide polymorphisms (SNPs), insertions, deletions, observed heterozygosity (Ho), and expected heterozygosity (He) counts in desi, kabuli, and wild populations.

	SNP	Insertions	Deletions	Ho	He
Desi	748,251	1,546,563	1,551,751	564,845	479,086
Kabuli	755,052	2,084,118	2,106,374	573,161	490,970
Wild	5,025,978	176,794	199,788	4,537,370	3,274,850

Synonymous and non‐synonymous changes in coding regions revealed a notable prevalence of deleterious mutations negatively impacting a higher number of genes within the wild chickpea population compared to the desi and kabuli populations. Conversely, synonymous changes were identified as neutral effective mutations occurring at a lower frequency compared to deleterious mutations across all three populations. Further analysis involved an annotation analysis of the non‐synonymous to synonymous substitution ratio (*ω*), allowing an assessment of the strength and mode of natural selection acting on protein‐coding genes in all three chickpea populations (Table 3). Notably, a higher occurrence of adaptive (diversifying) genes (*ω* > 1) was reported in the wild population (7992) in contrast to the desi and kabuli populations. The desi and kabuli populations exhibited comparable numbers of purifying genes (*ω* < 1), while the wild population displayed a significantly lower count. Also, these findings contribute to the understanding of evolutionary dynamics and selection pressures acting on protein‐coding genes in distinct chickpea populations, highlighting the prevalence of adaptive and purifying forces that shape genomic variations.

**TABLE 3:** The single nucleotide polymorphism (SNP) annotations and SNP count undergoing the selection pressure.

Population	Synonymous coding variants (dS)	Non‐synonymous coding variants (dN)	Positive selection genes (*ω* > 1)	Negative selection genes (*ω* < 1)	Neutral selection genes (*ω* = 1)
Desi	12,815	16,425	1454	2272	4157
Kabuli	12,945	16,605	1471	2282	4186
Wild	18,076	287,346	7,992	44	8111

*Note*: dS, synonymous; dN, non‐synonymous; *ω*, dN/dS ratio.

### 3.3 gPAV analysis

**FIGURE 2:**
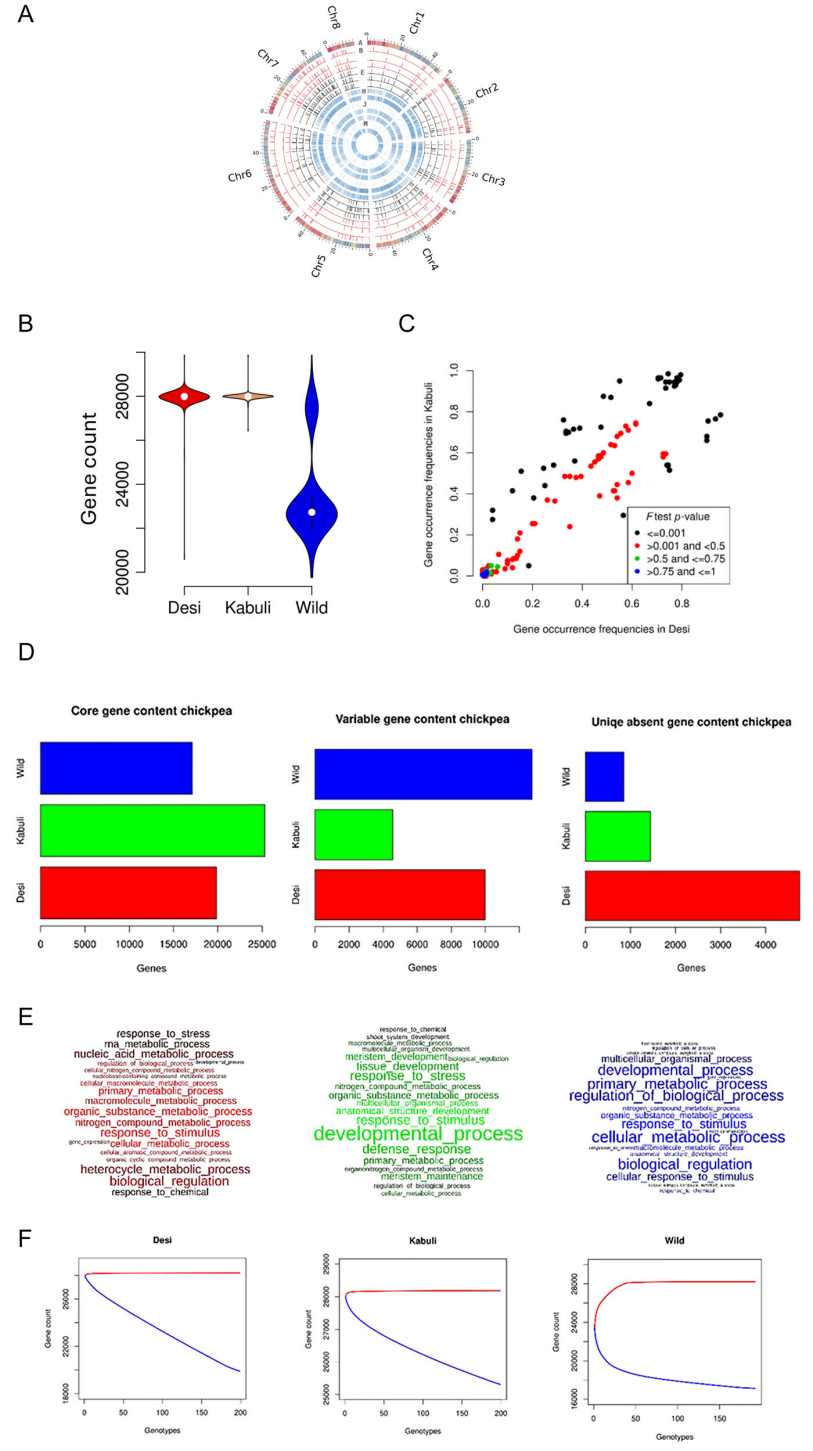
(A) Circos plot with track A representing the gene density of chickpea pangenome. The red color tracks (B, C, and D) correspond to the gene loss event reported in desi breeding lines, cultivars, and landraces. The green tracks (E, F, and G) correspond to the gene loss events in kabuli breeding lines, cultivars, and landraces. The blue tracks (H to N) represent the gene variations in wild species—*C. bijugum*, *C. cuneatum, C. echinospermum, C. judaicum, C. pinnatifidum, C. reticulatum*, and *C. yamashitae*. Plot B shows the gene count variability per accession in each chickpea population studied, and plot C indicates the gene occurrence frequency in comparison between desi and kabuli populations. The D plots show the housekeeping genes, variable genes, and uniquely absent genes, and the E plots represent the variable gene enrichment in desi, kabuli, and wild populations. The F plots represent the gene modeling with a number of genotypes (the red curve indicates the overall genes and the blue indicates the core/housekeeping genes) in desi, kabuli, and wild populations.

The WGRS data obtained from 593 accessions were mapped to the chickpea pangenome (Varshney et al., 2021). Gene PAVs were identified using the PAV analysis pipeline (Ruperao et al., 2021; Ruperao, Gandham, et al., 2023). A total of 16,117 (53.9%) dispensable genes across the 593 chickpea accessions were identified (Table S2) (Figure 2A). On assessing the individual chickpea population, 9993, 4566, and 12,749 dispensable genes were identified in desi, kabuli, and wild chickpea populations, respectively (Table S2). Though the overall gene count in both desi and kabuli populations is similar (Figure 2B), a high gene variability was observed in the desi compared to the kabuli population (Table S2). Further, gene comparison between desi and kabuli types resulted in a total of 49 significant genes (*p*‐value < 0.001) from the Fisher's test (Table S3) (Figure 2C). As expected, the wild population has more dispensable genes compared to desi and kabuli populations (Figure 2A,D). Comparing the gene count in each wild accession, the wild chickpea population can further be classified into two subgroups (Figure 2B). A group of 156 wild accessions having lower gene count ranging from 19,759 to 23,147 includes *Cicer cuneatum, Cicer yamashitae, Cicer judaicum, Cicer bijugum*, and *Cicer pinnatifidum* (wild group, WG1) (Table S4). The second wild group (WG2) comprises 37 accessions with gene count ranging from 27,085 to 28,111 and includes *Cicer echinospermum* and *Cicer reticulatum* (Table S4). Annotations suggest that these genes are enriched for functions associated with biological processes, cellular process proteins, response to defense, stimulus, stress, chemical, metabolic process, developmental process, and biological regulation (Figure 2E). A significant difference in the average number of genes was observed (Figure 2B). The kabuli population contains the largest number (27,988) of average genes (1882 ± 1569), followed by desi (27,924) (1946 ± 7342) and wild (23,499) (6371 ± 3740) populations (Figure 2B). The difference in the average gene number was due to the impact of the frequency of specific genes (Figure 2C). A total of 11 and 38 significantly different genes showed increased gene frequency in the desi and kabuli populations, respectively (Table S3). The number of uniquely absent and present genes was observed in the desi (4751; 13), kabuli (1442; 5), and wild population (4751; 15).

Gene enrichment analysis revealed that 134 variable genes were commonly enriched in all three populations. The wild population has the most unique (1170) enriched genes, followed by desi (662) and kabuli (16) populations (Figure S1). This indicates the difference in the gene content in different populations studied. Genes enriched in the top 20 GO terms were screened with differences in enrichment scores as candidate genes. Genes showing increased frequency are enriched in functions associated with disease resistance, protein kinase, copper ion transmembrane transporter, and flavin adenine dinucleotide (FAD)‐binding domain proteins (Table S3). Involvement of multiple molecular functions, biological processes, and cellular components was observed in GO terms, such as response to stimulus (GO:0050896), response to chemicals (GO:0042221), response to hormone (GO:0009725), system development (GO:0048731), plant organ development (GO:0099402), leaf development (GO:0048366), meristem development (GO:0048507), and maintenance (GO:0010073) (Figure S2). Finally, 1137, 322, and 1553 candidate genes from the desi, kabuli, and wild populations were enriched, totaling 2363 genes from the chickpea pangenome analysis (Figure S1).

### 3.4 GWAS analysis using multiple marker types

The GWAS analysis was performed independently using multiple marker types in this study to obtain stronger associations with key agronomic traits in chickpea. These traits include days to flowering (DTF, days), days to maturity (DTM, days), 100‐seed weight (HSW, g), plant height (PH, cm), and yield per plant (YPP, g). The analysis identified various types of genetic markers, encompassing SNPs, indels, and gPAVs. Significant markers associated with these traits were identified across all eight chickpea chromosomes (Chr1 to Chr8), employing two statistical methods, FarmCPU and MLM (Table 4). Further, the results showed both overlapping association signals across markers and unique associations specific to particular marker types.

**TABLE 4:** Genome‐wide marker‐trait association analysis with multiple marker types using multi‐model analysis.

Chromosome	Variations	Associated traits	Type	Methods	Marker types
Chr1	4	HSW, PH, DTM	Desi, kabuli	FarmCPU, MLM	SNP, gPAV
Chr2	1	DTM	Desi	FarmCPU	SNP
Chr3	5	HSW, PH	Desi	FarmCPU	SNP, indel
Chr4	4	HSW, PH	Desi	FarmCPU	SNP, indel
Chr5	6	DTF, DTM, HSW, YPP	Desi, kabuli	FarmCPU, MLM	SNP, indel, gPAV
Chr6	4	DTF, DTM	Desi	FarmCPU	SNP, indel
Chr7	10	DTF, DTM, PH	Desi	FarmCPU	SNP, gPAV
Chr8	4	DTF, HSW, PH, YPP	Desi, kabuli	FarmCPU, MLM	SNP, gPAV

Abbreviations: DTM, days to maturity; DTF; days to flowering; FarmCPU, fixed and random model circulating probability unification; gPAV, gene presence and absence variation; HSW, 100‐seed weight; indel, insertions and deletions; MLM, mixed linear model; PH, plant height; SNP, single nucleotide polymorphism; YPP, yield per plant.

In desi population, trait‐associated markers were identified on multiple chromosomes. The Chr2 (1) and Chr7 (10) have the minimum and maximum number of associated SNPs, respectively (Table 4). Four SNP markers were found on Chr5, Chr6, Chr7, and Chr8 for DTF. For DTM, 11 SNPs, one indel, and four gPAV markers were detected across various chromosomes. For the trait HSW, five SNPs and three indels were identified on Chr1, Chr3, Chr4, and Chr8, while for PH, eight SNPs were located on Chr1, Chr3, Chr4, Chr7, and Chr8. In the kabuli population, the analysis revealed one gPAV marker on Chr1 associated with DTM and four gPAVs on Chr5 and Chr8 associated with YPP.

Integrating SNPs and gPAVs in GWAS analysis is a powerful strategy to enhance comprehensiveness of genetic analysis for complex traits. The SNPs were the most prevalent marker type employed in this study. Nevertheless, gPAVs, representing the presence or absence of specific DNA sequence segments in different individuals or genotypes, were utilized to capture structural variations, such as gene duplications or deletions, which may contribute to phenotypic differences. The use of gPAVs is beneficial as they can capture a different type of genetic variation compared to SNPs and indels.

**FIGURE 3:**
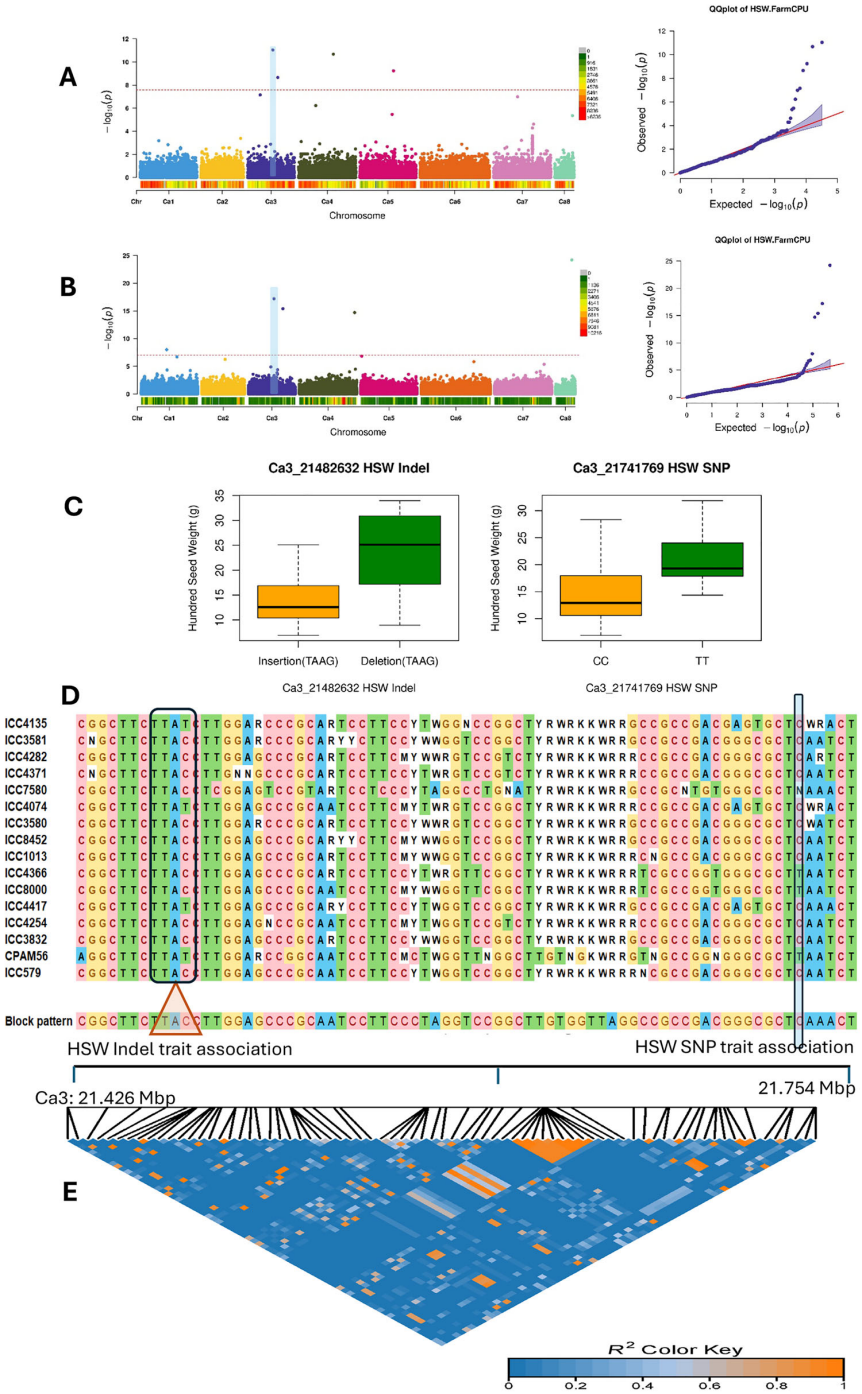
With the fixed and random model circulating probability unification (FarmCPU) method, the trait 100‐seed weight (HSW) shows association with (A) insertions and deletions (indels) and (B) single nucleotide polymorphism (SNP) markers, and (C) boxplot showing allelic effects of the peak indels and SNP markers associated with HSW in the chickpea desi population. (D) Showing the haplotypes (variant positions) between the two associated markers in the most promising chickpea accessions. (E) Genetic linkage between the markers in a 372 kb window.

In this study, multiple markers were associated with the trait HSW. For example, the SNP variation at Chr3: 21.7Mb (λGC 1.199) and the indel at Chr3: 21.4Mb (λGC 0.886) also showed association with the HSW trait (both marker's positions were 327 kb far apart, with more SNPs in this window range) (Figure 3), without any significant correlation between the markers. On assessing the haplotype patterns, 16 most promising accessions having similar patterns of alleles in this region were identified (Figure 3). Similarly, different traits associated with different markers at a distance of 397 kb were observed (Chr8:13369288:YPP and Chr8:13766972:HSW). Overall, 39 markers, including SNPs, indels, and gPAV, showed significant MTAs with different traits using the FarmCPU and MLM methods (Table S5). This GWAS analysis using multiple marker types provided valuable insights into the genetic markers associated with different agronomic traits in both desi and kabuli types. These markers can potentially be utilized in the marker‐assisted selection (MAS) of chickpea breeding programs.

### 3.5 Allelic effects of the trait‐associated markers

**FIGURE 4:**
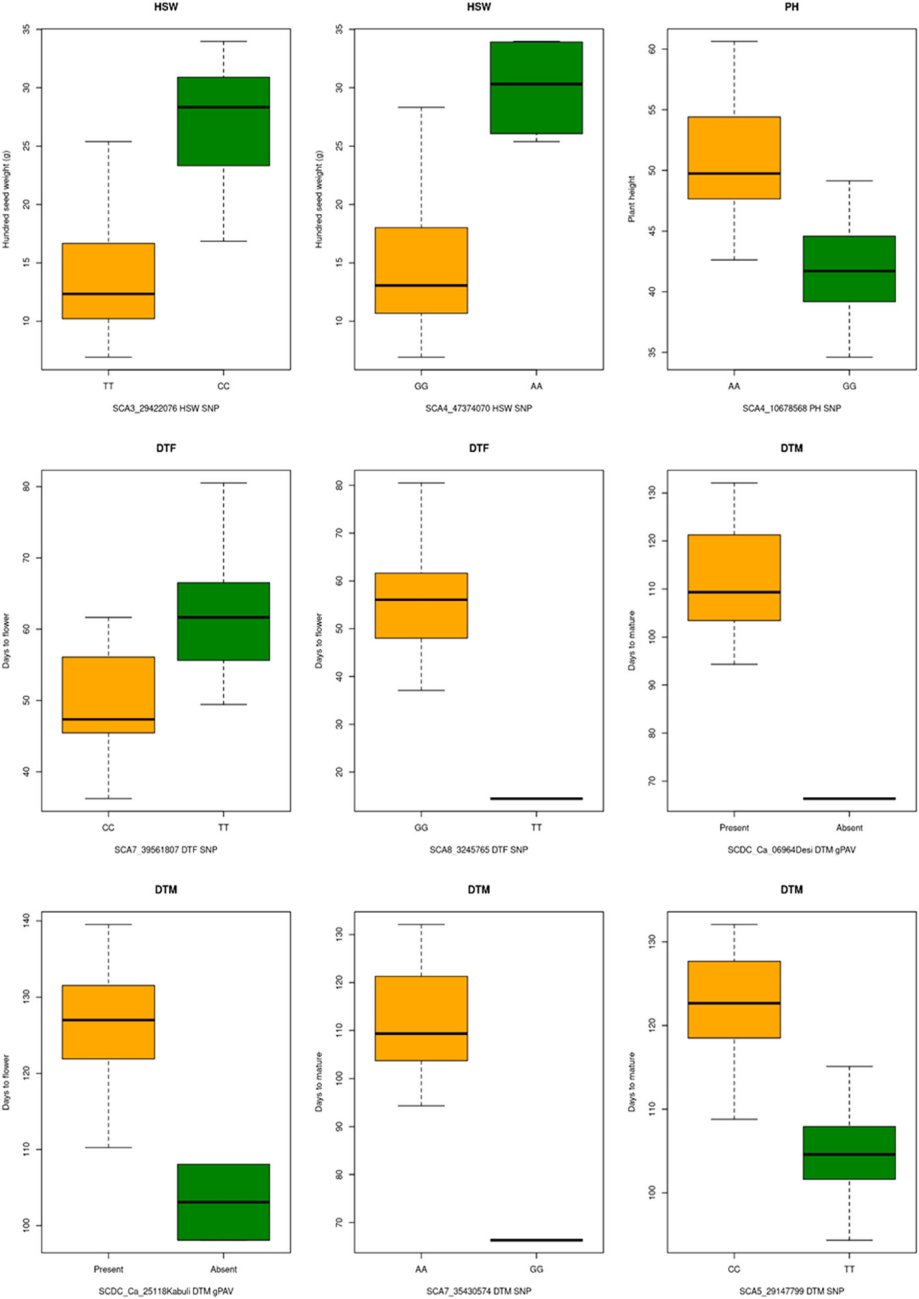
Boxplot showing allelic effects of the various markers (single nucleotide polymorphism [SNP], insertions and deletions [indels], and gene presence and absence variation [gPAV]) associated with 100‐seed weight (HSW), plant height (PH), days to flowering (DTF), and days to mature (DTM) traits based on the fixed and random model circulating probability unification (FarmCPU) and mixed linear model (MLM) genome‐wide association study (GWAS) models.

**FIGURE 5:**
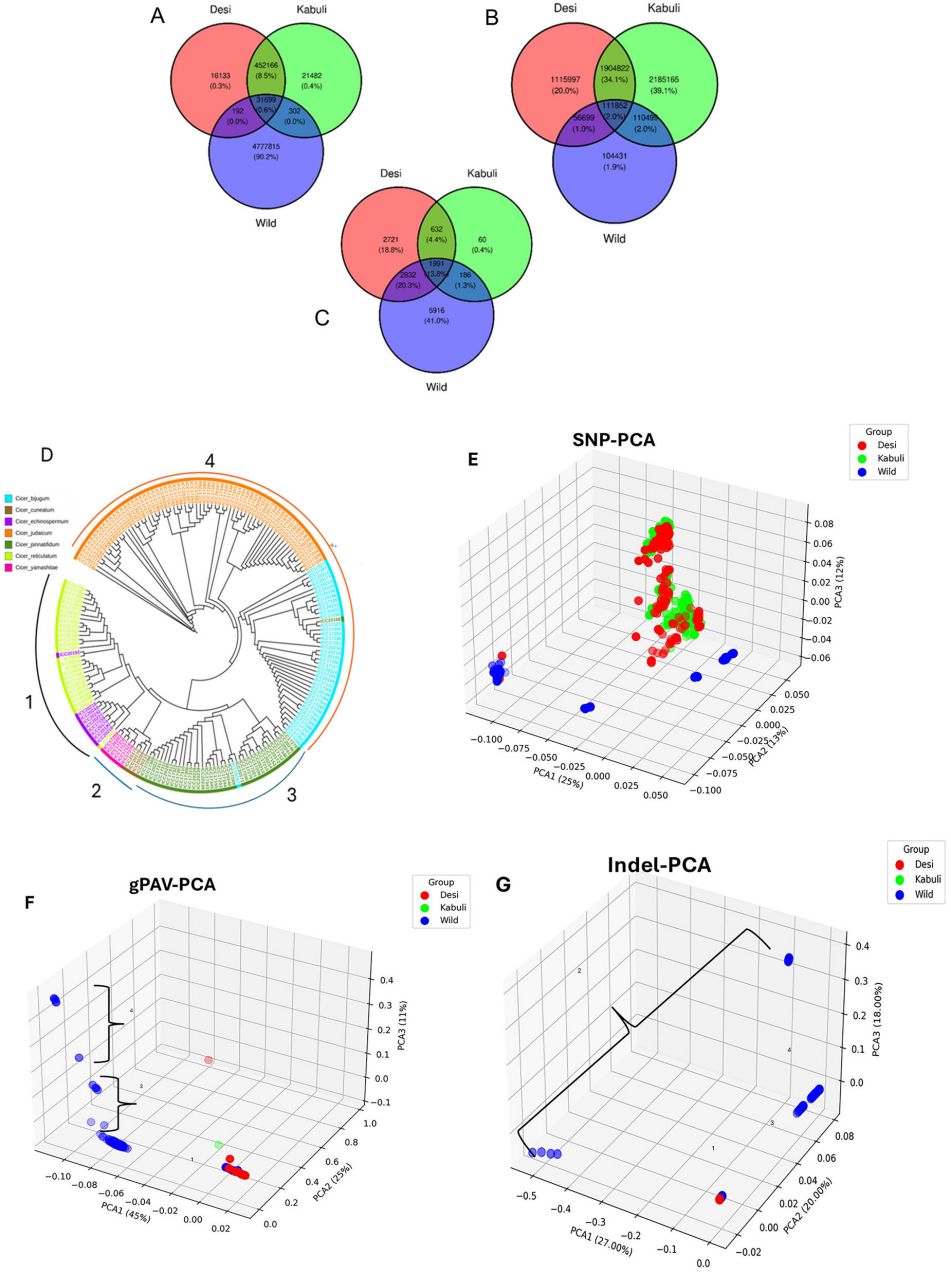
The (A) single nucleotide polymorphisms (SNPs), (B) insertions and deletions (indel), and (C) gene presence and absence variations (gPAVs) comparison between desi, kabuli, and wild populations. The phylogenetic relationship between the (D) wild accessions (including the seven subgroups) and principal component analysis (PCA) analysis of all three chickpea populations (desi, kabuli, and wild) with (E) SNPs, (F) gPAV, and (G) indels.

The allelic effects of the SNP markers show a clear differentiation in the expression. A total of nine markers (Table 4 and Table S5), which include five SNPs and four indels, were associated with the HSW. The “CC” allele of SNP marker SCA3_29422076 (with phenotypic variance explained [PVE] 7.49, Table S6) on chromosome Ca3 results in HSW (mean = 28 g) compared with a mean of 17 g among genotypes with the homozygous “TT” alternative allele (Figure 4). The HSW trait is also associated with the “AA” allele at SCA4_47374070 (PVE 12.87) with a mean weight of 30 g, whereas the “GG” allele showed a mean of 12.5 g (Figure 4). The PH was associated with eight SNPs (Table S5), and the mean of 50 cm length was recorded with the “AA” allele at SCA4_10678568 (PVE 1.67), and the minor allele “GG” was recorded with 42 cm of PH (Figure 4). For the DTF, four SNP markers (SCA5_29147799, SCA6_39401570, SCA7_39561807, and SCA8_3245765) on Chr 5, 6, 7, and 8 were associated with clear allelic difference. For example, SCA7_39561807 (PVE 3.68) SNP has the “CC” allele for the mean of 47 DTF and “TT” for a mean of 61 DTF. Comparatively, the mean difference between the “GG” (mean 56 days) and “TT” (mean 12 days) was high at the SCA8_3245765. A similar huge difference between the allelic effect was recorded for the trait DTM with 14 different markers (including eight SNPs, one indel, and five gPAVs). The two gPAV markers (SCDC_Ca_06964 and SCDC_Ca_25118) were absent in plants with early maturity (68–102 days), whereas delayed maturity (110–126 days) was recorded in plants having these genes (Figure 4). A similar difference was also seen with the allele variations. The alleles “AA” and “CC” for SCA7_35430574 (PVE 21.57) and SCA5_29147799 (PVE 13.37) SNP markers have delayed DTM, in the range of (mean) 110–122 days, respectively. However, the alternative alleles “GG” and “TT” are associated with a maturity range of 68–105 days (Figure 5). The advantage of using multiple marker types is demonstrated by the association of the YPP trait with four genes, where YPP increased to nearly 1200 g in accessions without these genes, compared to less than 200 g accessions with these genes.

Most of the trait associations with SNP and indel markers were seen in only desi population, but the gPAV has identified novel association with DTM and YPP (Figure S3). Among the 39 trait‐associated markers, most of the markers were identified using FarmCPU, while five markers (gPAVs) were identified with MLM. A high YPP was reported in genotypes IG71759 and ICC12461 with Ca17943, Ca01794, Ca08939, and Ca20114 absent in these accessions.

### 3.6 Genetic diversity insights within/between desi, kabuli, and wild populations

A comprehensive genetic analysis using various population genetics metrics was conducted. The even population datasets for each group help in understanding the genetic diversity of desi, kabuli, and wild accessions. We compared the level of genetic variations between desi, kabuli, and wild populations. Majority of SNP and gPAV variations, 90.2% and 41.0%, respectively, were reported as private variations from wild accessions. But this scenario has changed with the indel markers, which report only 1.9% private indels for wild accessions. Among the three markers used in this study, gPAVs showed a maximum of 13.8% (SNP 0.6% and indels 2.0%) of common genes (housekeeping genes) between the three populations. On comparing the desi with kabuli genetic variations, only 9.1% of SNPs, 36.1% of indels, and 18.2% of gPAVs are in common between these two populations. Indels are the most effective markers, with private indel markers highlighting the genetic uniqueness between the desi (20%) and kabuli (39.1%) populations (Figure 5). Additionally, a heterozygosity parameter was also assessed, which showed that the wild has the most (44.7%) private hets. Desi and kabuli share 48.6% of overall hets (Figure S4). We estimated expected heterozygosity (He), observed heterozygosity (Ho), and nucleotide diversity based on genotype frequencies (Table 5). The higher the population heterozygosity, the greater the population diversity. On comparison, we found that the wild pi (0.00409278) and the F_IS_ (0.907026) values were higher than the desi (pi = 0.000645363 and F_IS_ = 0.360453) and kabuli (pi = 0.000649127 and F_IS_ = 0.344156), and the value of observed heterozygosity (Ho) was always greater than that of expected heterozygosity (He) in each population. In addition, the genetic diversity of kabuli is higher than that of the desi, which indicates that in the desi (lower density) population, a long‐term artificial selection was used to create new hybrids for desi‐type chickpea improvement.

**TABLE 5:** The genetic diversity parameter measurement between the desi, kabuli, and wild populations.

Population	He	Ho	F_IS_	PA%	pi
Desi	0.667702	0.787224	0.360453	0.1	0.0006454
Kabuli	0.673236	0.78594	0.344156	0.2	0.0006491
Wild	0.702555	0.973406	0.907026	86.80%	0.0040928

*Note*: Ho, observed heterozygosity; He, expected heterozygosity; F_IS_, inbreed coefficient; PA%, percent of private alleles, and pi, nucleotide diversity (background color code: red for maximum, yellow for moderate, and green for minimum).

Additionally, the wild population being more diverse compared to desi and kabuli, the subgroup of *C. reticulatum* exhibits lower diversity (pi = 0.000608254) compared to the kabuli group (pi = 0.000649127). Within each desi and kabuli group, we found that the kabuli lines and landraces have higher diversity (pi KC = 0.000999325 and KL = 0.000679136) value, and the F_IS_ in kabuli has a higher value in kabuli landrace (0.349629) and decreased in the cultivars, whereas the F_IS_ desi subgroups report a higher F_IS_ for the desi cultivars (desi F_IS_ = 0.351521) (Tables 5 and 6). The SNP density, transition, transversion, indels, and heterozygosity parameters also differ between and within populations. Compared to the desi and kabuli populations, the wild population has higher SNP density, indels, and heterozygosity (Figure S5). Among the kabuli population, the heterozygosity is higher in the landraces and least in the cultivars. The desi population showed higher gene variability compared to the kabuli population (Figure S5). However, the difference between the desi and kabuli populations is very small. In the desi population, the landraces have more diversity than breeding lines, followed by the cultivars, whereas, in kabuli, the cultivars have more diversity than the landraces and are followed by breeding lines. This may be because both desi landraces and kabuli cultivars have undergone long‐term artificial selection for quality traits.

**TABLE 6:** The detailed genetic diversity parameter measurement within the desi, kabuli, and wild populations (between the subgroups).

Population	He	Ho	F_IS_	pi
DL	0.667029	0.782974	0.347723	0.00065844
DC	0.667889	0.792377	0.351521	0.00054305
DB	0.636472	0.75959	0.322699	0.00065512
KL	0.662947	0.781282	0.349629	0.00067914
KC	0.379158	0.615584	0.254013	0.00099933
KB	0.66738	0.770306	0.295432	0.00065203
WG1	0.71291	0.960706	0.859265	0.00323115
WG2	0.671091	0.957237	0.849012	0.00060825

*Note*: Ho, observed heterozygosity; He, expected heterozygosity; F_IS_, inbreed coefficient; PA%, percent of private alleles; and pi, nucleotide diversity (background color code: red for maximum, yellow for moderate, and green for minimum).

Abbreviations: DB, desi breeding lines; DC, desi cultivar lines; DL, desi landrace lines; KB, kabuli breeding lines; KC, kabuli cultivar lines; KL, kabuli landrace lines; WG1, wild group 1; WG2, wild group 2.

The genome‐wide analysis of genetic differentiation between the desi and kabuli populations revealed a total of 973 genes with high F_ST_ values and 7014 genes with low F_ST_ values (Table S7). High F_ST_ values indicate a substantial degree of genetic differentiation or restricted gene flow between the desi and kabuli populations for these genes (Figure S6). This observation suggests a limited exchange of genetic material for these specific genes across the populations. The elevated F_ST_ values for these genes may indicate that they are under diversifying selection or local adaptation, where different populations have evolved to adapt to unique environmental conditions or selective pressures. Notably, the genes exhibiting high fixation indices are primarily involved in membrane and transporter activities, DNA‐binding, ion‐binding transcription factors, flower development, response to symbiotic fungi, adenosine triphosphate (ATP) hydrolysis activity, defense response, and response to oxygen‐containing compounds or chemical stimuli (Table S7).

In contrast, low F_ST_ values recommend a high degree of genetic similarity or low differentiation between the desi and kabuli populations for these 7014 genes. This observation suggests a substantial gene flow or shared ancestry between the populations for these genes, implying that the populations are not genetically isolated from each other for these genomic regions. The low F_ST_ values observed for these genes suggest that they are under purifying selection or play crucial roles in essential biological processes, thus constraining genetic variation. The genes with low F_ST_ values are primarily involved in ion transmembrane transporter activity, protein kinase activity, ATP binding, potassium channel activity, RNA binding, rRNA processing, protein transport, defense response, response to other organisms, response to stimuli, DNA repair, ion binding, and cell division (Table S7).

The genes exhibiting high F_ST_ values, which are associated with local adaptation and population differentiation, can be valuable for breeding programs, conservation efforts, and understanding the evolutionary dynamics of plant populations. On the other hand, the genes with low F_ST_ values may represent conserved genomic regions essential for plant survival and could serve as potential targets for genetic engineering or biotechnological applications where the conservation of gene function is desired across diverse plant populations or species.

The Euclidean genetic distance‐based neighbor‐joining tree shows mixed accessions (landraces, cultivars, and breeding lines) in both the desi and kabuli populations (Figures S7 and S8), whereas the wild population shows the cluster of *C. reticulatum* and *C. echinospermum* (cluster 1) and the other three clusters (Figure 5). The results of phylogenetic tree analysis showed that the desi and kabuli accessions were mixed into the same genetic groups, confirming their genetic relatedness. Due to the geographical proximity and the low degree of differentiation of the desi and kabuli populations, there is a gene flow between the two groups.

The PCA results clearly distinguish the wild population with desi and kabuli chickpea types (Figure 5). Among the PCAs with three marker systems, the SNPs and gPAVs exhibit clear wild subgroups more than the indel PCA (Figure 5 and Figure S9). The three marker types (SNP, indel, and gPAV) were used for assessing the PCA between the subgroups. The first three principal components explained 51.80%, 66.80%, and 81.44% of the total variance with SNPs, indels, and gPAVs markers, respectively, and were used to visualize the relationship between the chickpea populations.

## 4 DISCUSSION

Diversity analysis and GWAS are widely used to identify diverse gene pools and map genetic variants linked to traits of interest, respectively. However, the reliability of such studies can be influenced by sample composition, marker type, and statistical methods. Use of multiple marker types results in a comprehensive diversity analysis and GWAS in crops like chickpea with narrow genetic base. Limited studies have reported diversity and GWAS analysis using multiple marker types. Hence, the current study employed the multiple marker strategy to demonstrate the effectiveness of multiple marker types in genetic diversity and MTA analysis across different chickpea populations—desi, kabuli, and wild species. For this to be achieved, an adequate sample size within and between populations is essential. The concept of optimal sample size resides in the fact that it correctly defines and estimates the genetic variance in the studied chickpea populations. Populations may carry low‐frequency genetic variants or alleles that can go unnoticed or underrepresented if the populations’ sample size is small. A sample with a higher number of individuals raises the probability of identifying such rare variants because they may be the genetic makeup of the whole population (Zuk et al., 2014). Gene frequencies are the basis of population genetics and an important tool to estimate genetic variability (Greenbaum et al., 2014). While analyzing the small sample size, the probability of observing some alleles may be different from the true probability of their occurrence in populations. Therefore, the estimated allele frequencies may be biased or involve large variances, thus yielding a poor estimate of the genetic diversities. Furthermore, it is crucial to meet the representativeness of the samples when genetic diversity is investigated. Related to this is the dependence on small sample sizes that are likely to provide a poor picture of the genetic stock of the whole population by virtue of under‐representative data (Bashalkhanov et al., 2009). The number of around 200 accessions from each of the three populations—desi, kabuli, and wild—drawn from a larger panel are used for capturing the maximum amount of genetic variation within chickpea species.

### 4.1 Multi‐marker GWAS analysis

The application of SNPs is useful because it is bi‐allelic and co‐dominant and, thus, can be genotyped using high‐throughput systems. SNPs are ideal for GWAS so that a large number of variations are obtained throughout the population. It is, however, noteworthy that indels, although less represented, were detected for many desirable traits in crops. This study identified association of indels for the traits DTM and HSW in the desi population. Non‐synonymous indels can individually affect the gene and therefore the phenotype, and hence act as a putative source of phenotypic variation while providing additional information orthogonal to that obtained from common GWAS. Hence, wild accessions had the largest number of SNPs, 5,025,978 and the least number of indels, 319,471, indicating point mutation as being more frequent than insertions or deletions as reported in Thudi et al. (2016). The desi and kabuli accessions had a smaller total of SNPs (748,251 and 755,052), but a large number of indels (2,307,530 and 3,134,439), which suggests that more indels exist in the cultivated chickpeas. The transversion mutation/transition mutation (Tv/Ts) ratio was lower in wild population (1.39) than that of desi (1.66) and kabuli (1.64), or the autochthonous population, indicating a higher frequency of transition above and beyond that of transversion. The wild accessions had a higher proportion of private indels of 1.9%, while desi and kabuli showed 20% and 39%, respectively, suggesting a higher level of indel polymorphisms in the wild population. The increased value of nucleotide diversity (*π*) in the wild population compared with the cultivated chickpeas, hence, supports the previous studies (Getahun et al., 2021; Singh et al., 2022). Earlier studies report that the domestication and breeding have significant effects on genetic variation of chickpeas. Furthermore, desi and kabuli accessions have been through bottlenecks and selection, thus carrying less genetic variation than wild relatives (Varshney et al., 2021). The comparisons of SNP, indel, and Tv/Ts ratios give crucial information on the mutational and evolutionary mechanisms that have an impact on the genetic constitution of these chickpea populations.

Out of the total 29,854 genes, 16,117 (53.9%) were considered dispensable genes in 593 chickpea accessions in the current study, where wild population had a maximum of 12,749 dispensable genes followed by desi 9993 and kabuli 4566. Despite the fact that both desi and kabuli have approximately the same gene number, gene variability in desi accessions is higher than in the kabuli population. The wild accessions were further classified into two subgroups based on gene count: WG1 having 156 accessions with 19,759–23,147 genes and WG2 having 37 accessions with 27,085–28,111 genes. The dispensable genes are associated with biological functions that are ranked by GO terms—biological process, cellular component, and molecular functions—with defense response, metabolic process, and development. The gene number with the mean value of this feature is also highest for kabuli (27,988), followed by desi (27,924) and lowest for wild (23,499) accessions. Some of the gene frequency gaps correspond to the gene expression means, with 11 and 38 genes showing higher frequencies in desi and kabuli, respectively. The wild population contained the largest number and proportion of the genes (1170) under expansion, followed by the desi (662) and kabuli (16) populations. Most of the frequently observed genes were associated with disease resistance, kinases, ion transport, FAD‐binding domain proteins, and so forth. Further, additional molecular functions, biological processes, and cellular components are recognized (Table S8).

For the traits of interest in the desi accessions, we identified the presence of causative markers in several chromosomes, and among them, SNP was the most commonly identified marker type. For example, four SNP markers were found for DTF and 11 SNP markers, one indel, and four gPAV markers were detected for DTM, and these were located across different chromosomes. More specifically, in HSW, both SNPs and indels were found to reside on distinct chromosomes for HSW and PH. Kabuli accessions exhibited fewer markers, whereby one Chr1 gPAV marker was linked to DTM and four gPAV markers on Chr5 and Chr8 to YPP. These findings point to the fact that the choice of markers comes into play while performing GWAS analysis, and this should involve the use of markers that give different aspects of genetic diversity. Therefore, by combining the studied types of genetic variation, inclusive of SNPs, indels, and gPAVs, this research increases the probability of identifying suitable markers associated with the considered traits and provides a broader perception of the genetic architecture that controls complex traits in chickpea. The main findings of this GWAS exhibit clear genomic distinctions between desi and kabuli chickpeas, which implies the genetic disparity in desi chickpea (Istanbuli et al., 2024). Combination of marker types was tested for the GWAS of agronomic traits such as DTF, DTM, HSW, PH, and YPP in the present study based on the fact that they indicate various aspects of genetic variation. Moreover, different types of markers can influence gene function and expression at different levels, enabling the determination of potential regulative or causative variants/markers in linkage with causal variants. The use of both complementary forms of markers and multi‐model analysis can improve the ability and precision of the GWAS, which will ultimately improve the understanding of the genetics of the crop. Hence, the results provided fundamental comparative genetic features associated with the traits on all chickpea chromosomes from Ca1 to Ca8, which are in addition to the prior investigation (Varshney et al., 2021).

### 4.2 Genetic diversity in wild populations

SNPs and gPAVs discovered in wild chickpeas were classified as private variations, of which 90% belonged to wild chickpeas with 2% and 41.0%, respectively. However, for indels one parameter is calculated, which is the time to double, that is, the doubling time work done under the previous regulation was twice as fast as that done under the new regulation. About 9% of wild accessions were private. Higher ratios of private variations are noticed in wild populations, which is a sign of a wider gene pool, which in turn provides more genetic plasticity to the populations.

Population structure and diversity analyses revealed key contrasts between wild and cultivated chickpea. The optimal K‐values differed across groups, with kabuli showing *K* = 10, desi *K* = 9, and wild *K* = 6, reflecting distinct genetic stratification patterns. Diversity parameters also highlighted strong contrasts: wild chickpea displayed the highest heterozygosity (Ho = 0.973) and nucleotide diversity (*π* = 0.0041), alongside a remarkably high proportion of private alleles (PA = 86.8%), compared to desi (Ho = 0.787, *π* = 0.00065, and PA = 0.1%) and kabuli (Ho = 0.786, *π* = 0.00065, and PA = 0.2%). The F_ST_‐based comparisons further confirmed this divergence, with wild accessions harboring substantially more variable genes (158 high‐F_ST_ and 1803 low‐F_ST_) than desi (157 high and 1042 low) and kabuli (41 high and 290 low). These results demonstrate that wild relatives are highly diverged from cultivated chickpea, maintaining a broader genetic reservoir shaped by long evolutionary adaptation. Many genes absent in cultivated lines but retained in wild germplasm represent critical resources for breeding, offering potential to enhance yield, resilience, and adaptation in future chickpea improvement programs.

A detailed comparison across the high and low F_ST_ annotated gene lists for desi, kabuli, and wild chickpea types revealed important patterns supporting broader biological relevance. Notably, several well‐known disease resistance gene families, such as nucleotide binding site‐leucine rich repeat and RING‐type E3 ubiquitin transferases, appear repeatedly among high F_ST_ wild and differentiated presence/absence lists, underscoring increased biotic stress resistance in wild chickpea. Flowering time and maturity regulators, including FRIGIDA‐like and GATA transcription factor, emerge in desi and kabuli sets, absent or differentiated in some contrasts, providing probable links to domestication and adaptation. Genes associated with abiotic stress (e.g., heat shock proteins, and aquaporins) and hormonal regulation (e.g., ethylene responsive factors and gibberellin dioxygenases) are also prominent, especially among desi and kabuli sets. These findings highlight clear connections between genetic differentiation, domestication traits, and breeding targets for chickpea improvement.

### 4.3 Commonality among desi, kabuli, and wild populations

The housekeeping genes that are common among three groups of desi, kabuli, and wild populations are 13.8% by gPAVs, 0.6% by SNPs, and 2.0% by indels. In desi and kabuli populations, 1% of SNPs and 36.8% of deletions were located on intronic regions. Further, 2% of gPAVs were shared between the two populations. It can be concluded that a high proportion of indels in desi (20%) and kabuli (39.1%) are actual mutations; hence, the obtained results are genetically distinctive.

### 4.4 Heterozygosity and genetic variability

Values for the heterozygosity measures revealed that wild populations possessed the highest (44.7%) amount of private heterozygosity followed by desi and kabuli having 48.6%. This implies that the wild chickpea group has a wider base in terms of genetic improvement and thus will be able to cope with environmental changes (Singh et al., 2022). In the comparison between Ho and He, these values were significantly different; Ho was higher compared to the expected level of He, which prompted the conclusion that the amount of genetic variability within the populations was rather high.

### 4.5 Genetic distance and PCA

Our results demonstrated that both desi and kabuli populations had a mixture of landraces, cultivars, and breeding lines, and hence there was gene flow between these two groups. Similar observations were reported in previous chickpea studies (Thudi et al., 2016). However, in the case of the wild population, the structure was distinguishable undoubtedly because of the background of the population.

It was evident from PCA results that the chickpea types were grouped as wild and domesticated. The identified PCs for the analysis were the first three, which accounted for 51.80%, 66.80%, and 81%. Of the total variability accounted for by SNP, indel, and gPAV, the three accounted for 44% each. The SNPs and gPAVs displayed better subdivisions in the wild population, which implied that more genetic variation existed in wild population as compared to the desi and kabuli populations. In general, it can be concluded that wild chickpea has a higher level of genetic differentiation than desi and kabuli populations, some of which have unique genetic signatures and new genes; therefore, the observed gene flows make a more complicated population structure (Varshney et al., 2021). This genetic difference is essential for knowing the possibilities of breeding and evolution in chickpea.

This study provides a broad picture and comprehensive understanding of genetic basis of complex traits. Overall, due to the high‐frequency rate of SNPs as a form of genetic variation, several traits have high‐resolution maps, including in chickpea (Barmukh et al., 2021). Due to their dispersed nature, they are particularly useful for mapping both large‐effect and small‐effect quantitative trait loci. The indels and gPAVs are less common as compared to SNPs but are capable of causing major functional changes to the genes and variation in the phenotypes, making them useful in supplementing the information from the SNPs. Also, it makes MAS more precise, increasing the effectiveness of the breeding programs. This multi‐marker approach enhances the ability of understanding complex traits such as yield, yield component traits, drought tolerance, and so forth, which are all major concerns not only for chickpea or legumes but for crop production as a whole with the changing climate patterns across the globe.

Second, the requirement of a large amount of germplasm resources is critical for identifying genetic resources to enhance crop traits such as disease resistance and nutritional quality (Jukanti et al., 2012). This is particularly important in crops like chickpea, which are among the most important nutritious legumes consumed worldwide. Further, with an understanding of the genetics of quantitative traits, these technologies are set to become more useful in legume breeding programs. In the context of the study, the findings give useful information about the potential of employing these marker resources to advance legumes as well as crop breeding programs.

## 5 CONCLUSIONS

Thus, using SNPs, indels, and gPAVs in GWAS has significantly transformed the approach to improving chickpea varieties. Such comprehensive approaches to genetic analysis have proven very useful in understanding the genetic relations governing issues of complex genetics in chickpea populations. Key findings from the study are as follows: (a) The wild chickpea population exhibits the highest genetic diversity, with 90.2% of SNPs and 41.0% of gPAVs identified as private SNPs. (b) Desi and kabuli populations showed varying degrees of unique genetic markers supporting distinct evolutionary paths. (c) The observed heterozygosity was more significant than expected, which indicates high genetic diversity. (d) GWAS identified genetic markers linked to agronomic traits across all chickpea chromosomes, providing potential targets for MAS.

Therefore, employing multiple marker types in genetics research is crucial to encompass different manifestations of genetic differences. SNPs, indels, and gPAVs serve as effective resources for diversity analysis and GWAS, contributing significantly to chickpea improvement. They also open opportunities for further understanding complex traits such as drought tolerance and disease resistance. The documented genetic variation, especially the one assessed in wild populations, may be useful for future breeding programs driven by climate change. As more of the genetics underlying complex traits in chickpea are discovered, these technologies will become increasingly important for genomics‐assisted selection in crop breeding. In future research, these data shall be used in actual breeding processes involving relevant marker types mentioned for desired trait enhancement. Way forward, emphasis should be on the functional relationships of these identified genetic variations to add deeper knowledge about the genetics feeding into breeding efforts for the development of superior, climate‐resilient, and high‐yielding chickpea varieties.

## AUTHOR CONTRIBUTIONS

**Pradeep Ruperao**: Data curation; formal analysis; software; writing—original draft; writing—review and editing. **Anil Kumar Vemula**: Formal analysis; investigation. **Vinod Kumar Valluri**: Software. **Sriswathi Manda**: Project administration; writing—review and editing. **Rakesh K. Srivastava**: Writing—review and editing. **Sean Mayes**: Funding acquisition; resources; supervision. **Rajeev K. Varshney**: Funding acquisition; resources; writing—review and editing. **Himabindu Kudapa**: Conceptualization; funding acquisition; investigation; resources; writing—review and editing.

## CONFLICT OF INTEREST STATEMENT

The authors declare no conflicts of interests.

## Supporting information




**Supplementary Figure 1**: Gene Enrichment Analysis of Genes Among Chickpea Types: This figure illustrates the gene enrichment analysis of shared and unique genes between desi, kabuli, and wild chickpea types.
**Supplementary Figure 2: a**) Gene Ontology Enrichment Analysis of Biological Processes in Desi Chickpea Enriched Genes: This figure illustrates the Gene Ontology (GO) enrichment analysis of biological processes associated with genes enriched in desi chickpea.
**Supplementary Figure 2: b**) Gene Ontology Enrichment Analysis of Molecular Functions in Desi Chickpea Enriched Genes: This figure illustrates the Gene Ontology (GO) enrichment analysis focusing on molecular functions associated with genes enriched in desi chickpea.
**Supplementary Figure 2: c**) Gene Ontology Enrichment Analysis of Cellular Components in Desi Chickpea Enriched Genes: This figure illustrates the Gene Ontology (GO) enrichment analysis focusing on cellular components associated with genes enriched in desi chickpea.
**Supplementary Figure 2: d**) Gene Ontology Enrichment Analysis of Biological Processes in Kabuli Chickpea Enriched Genes: This figure illustrates the Gene Ontology (GO) enrichment analysis focusing on biological processes associated with genes enriched in kabuli chickpea.
**Supplementary Figure 2: e**) Gene Ontology Enrichment Analysis of Molecular Functions in Kabuli Chickpea Enriched Genes: This figure illustrates the Gene Ontology (GO) enrichment analysis focusing on molecular functions associated with genes enriched in kabuli chickpea.
**Supplementary Figure 2: f**) Gene Ontology Enrichment Analysis of Cellular Components in Kabuli Chickpea Enriched Genes: This figure illustrates the Gene Ontology (GO) enrichment analysis focusing on cellular components associated with genes enriched in kabuli chickpea.
**Supplementary Figure 2: g)** Gene Ontology Enrichment Analysis of Biological Processes in Wild Chickpea Enriched Genes: This figure illustrates the Gene Ontology (GO) enrichment analysis focusing on cellular components associated with genes enriched in wild chickpea.
**Supplementary Figure 2: h)** Gene Ontology Enrichment Analysis of molecular function in Wild Chickpea Enriched Genes: This figure illustrates the Gene Ontology (GO) enrichment analysis focusing on molecular function associated with genes enriched in wild chickpea.
**Supplementary Figure 2: i)** Gene Ontology Enrichment Analysis of Cellular Component in Wild Chickpea Enriched Genes: This figure illustrates the Gene Ontology (GO) enrichment analysis focusing on cellular components associated with genes enriched in wild chickpea.
**Supplementary Figure 3**: The box plot shows the gPAV marker effect on the yield per plant (YPP) trait association with a mixed linear model genome‐wide association study.
**Supplementary Figure 4**: The heterozygosity comparison between desi, kabuli and wild populations.
**Supplementary Figure 5**: A comparative measure of Ts/Tv, Indels, Heterozygosity, and variable genes between the chickpea desi, kabuli and wild sub‐groups.
**Supplementary Figure 6**: The genome wild F_ST_ measure, a density comparison between all the chickpea chromosomes.
**Supplementary figure 7**: Desi population NJ analysis show the genetic relationship between the breedinglines, cultivars and landraces.
**Supplementary Figure 8**: Kabuli population NJ analysis shows the genetic relationship between Kabuli breeding lines, cultivars, landracea, and the unknown types.
**Supplementary Figure 9**: The PCA analysis of SNPs (A) and gPAV (B) showing the relationship between the desi, kabuli and wild sub‐groups.

**Supplementary Table 1**: Chickpea diverse desi, kabuli and wild accessions.
**Supplementary Table 2**: The list of variable genes present in different populations.
**Supplementary Table 3**: Fisher test in highly variable genes.
**Supplementary Table 4**: Gene count in wild accessions and its subgroups.
**Supplementary Table 5**: Significant markers showing the MTA's.
**Supplementary Table 6**: Phenotypic variance explained for traits associated SNP markers.
**Supplementary Table 7**: High and low fixation index (F_ST_) of chickpea genes.
**Supplementary Table 8**: Chickpea pangenome genes annotations.

## Data Availability

The datasets used in this study are available at NCBI Sequence Read Archive database: PRJNA657888 (Varshney et al., 2021). The VCF files, PAV matrix, and analysis scripts have been made available at https://cegresources.icrisat.org/data_public/Chickpea_Rpradeep/.
