# Supplementary material for: Assessing genetic diversity and performing genome‐wide association studies (GWAS) using multiple marker types across desi, kabuli, and wild accessions in chickpea (Cicer arietinum L.)
Source: Plant Genome. 2025 Nov 23;18(4):e70149. doi: 10.1002/tpg2.70149 (PMC12640892; doi:10.1002/tpg2.70149)
Supplement: Supplementary file 1 [file TPG2-18-e70149-s002.docx]

**Supplementary Figure 1:** Gene Enrichment Analysis of Genes Among Chickpea Types: This figure illustrates the gene enrichment analysis of shared and unique genes between desi, kabuli, and wild chickpea types. The analysis reveals the distribution and functional significance of common genetic elements across these diverse chickpea populations

**Supplementary Figure 2:** a) Gene Ontology Enrichment Analysis of Biological Processes in Desi Chickpea Enriched Genes: This figure illustrates the Gene Ontology (GO) enrichment analysis of biological processes associated with genes enriched in desi chickpea. The analysis reveals key biological functions that are overrepresented in the desi chickpea genome.

**Supplementary Figure 2: b)** Gene Ontology Enrichment Analysis of Molecular Functions in Desi Chickpea Enriched Genes: This figure illustrates the Gene Ontology (GO) enrichment analysis focusing on molecular functions associated with genes enriched in desi chickpea. The analysis reveals key functional roles that are overrepresented in the desi chickpea genome.

Supplementary Figure 2: c) Gene Ontology Enrichment Analysis of Cellular Components in Desi Chickpea Enriched Genes: This figure illustrates the Gene Ontology (GO) enrichment analysis focusing on cellular components associated with genes enriched in desi chickpea. The analysis reveals key subcellular localizations that are overrepresented in the desi chickpea genome

**Supplementary Figure 2: d)** Gene Ontology Enrichment Analysis of Biological Processes in Kabuli Chickpea Enriched Genes: This figure illustrates the Gene Ontology (GO) enrichment analysis focusing on biological processes associated with genes enriched in kabuli chickpea. The analysis reveals key biological functions that are overrepresented in the kabuli chickpea genome.

**Supplementary Figure 2: e)** Gene Ontology Enrichment Analysis of Molecular Functions in Kabuli Chickpea Enriched Genes: This figure illustrates the Gene Ontology (GO) enrichment analysis focusing on molecular functions associated with genes enriched in kabuli chickpea. The analysis reveals key functional roles that are overrepresented in the kabuli chickpea genome.

**Supplementary Figure 2: f)** Gene Ontology Enrichment Analysis of Cellular Components in Kabuli Chickpea Enriched Genes: This figure illustrates the Gene Ontology (GO) enrichment analysis focusing on cellular components associated with genes enriched in kabuli chickpea. The analysis reveals key subcellular localizations that are overrepresented in the kabuli chickpea genome.

**Supplementary Figure 2: g)** Gene Ontology Enrichment Analysis of Biological Processes in Wild Chickpea Enriched Genes: This figure illustrates the Gene Ontology (GO) enrichment analysis focusing on cellular components associated with genes enriched in wild chickpea. The analysis reveals key subcellular localizations that are overrepresented in the wild chickpea genome.

**Supplementary Figure 2: h)** Gene Ontology Enrichment Analysis of molecular function in Wild Chickpea Enriched Genes: This figure illustrates the Gene Ontology (GO) enrichment analysis focusing on molecular function associated with genes enriched in wild chickpea. The analysis reveals key subcellular localizations that are overrepresented in the wild chickpea genome.

**Supplementary Figure 2: i)** Gene Ontology Enrichment Analysis of Cellular Component in Wild Chickpea Enriched Genes: This figure illustrates the Gene Ontology (GO) enrichment analysis focusing on cellular components associated with genes enriched in wild chickpea. The analysis reveals key subcellular localizations that are overrepresented in the wild chickpea genome

**Supplementary Figure 3**: The box plot shows the gPAV marker effect on the yield per plant (YPP) trait association with a mixed linear model genome-wide association study.

|  |   **Supplementary Figure 4:** The heterozygosity comparison between desi, kabuli and wild populations.    **Supplementary Figure 5**: A comparative measure of Ts/Tv, Indels, Heterozygosity, and variable genes between the chickpea desi, kabuli and wild sub-groups.      **Supplementary Figure 6:** The genome wild Fst measure, a density comparison between all the chickpea chromosomes.    **Supplementary figure 7:** Desi population NJ analysis show the genetic relationship between the breedinglines, cultivars and landraces.    **Supplementary Figure 8:** Kabuli population NJ analysis shows the genetic relationship between Kabuli breeding lines, cultivars, landracea, and the unknown types. |
| --- | --- |
| A |  |
| B |  |

**Supplementary Figure 9:** The PCA analysis of SNPs (A) and gPAV (B) showing the relationship between the desi, kabuli and wild sub-groups.
